# Impact of Genetic Variations in HIV-1 Tat on LTR-Mediated Transcription via TAR RNA Interaction

**DOI:** 10.3389/fmicb.2017.00706

**Published:** 2017-04-21

**Authors:** Larance Ronsard, Nilanjana Ganguli, Vivek K. Singh, Kumaravel Mohankumar, Tripti Rai, Subhashree Sridharan, Sankar Pajaniradje, Binod Kumar, Devesh Rai, Suhnrita Chaudhuri, Mohane S. Coumar, Vishnampettai G. Ramachandran, Akhil C. Banerjea

**Affiliations:** ^1^Laboratory of Virology, National Institute of ImmunologyDelhi, India; ^2^Department of Microbiology, University College of Medical Sciences and Guru Teg Bahadur HospitalDelhi, India; ^3^Centre for Bioinformatics, School of Life Sciences, Pondicherry UniversityPondicherry, India; ^4^Department of Biochemistry and Molecular Biology, Pondicherry UniversityPondicherry, India; ^5^Department of Veterinary Physiology and Pharmacology, Texas A&M University, College StationTX, USA; ^6^Department of Gastroenterology and Human Nutrition, All India Institute of Medical SciencesDelhi, India; ^7^Department of Symptom Research, The University of Texas MD Anderson Cancer Center, HoustonTX, USA; ^8^Department of Microbiology and Immunology, Rosalind Franklin University of Medicine and Science, ChicagoIL, USA; ^9^Department of Microbiology, All India Institute of Medical SciencesDelhi, India; ^10^Department of Neurological Surgery, Northwestern University, ChicagoIL, USA

**Keywords:** HIV-1 Tat, transactivation, TAR RNA, genetic variations, recombination, mutations

## Abstract

HIV-1 evades host defense through mutations and recombination events, generating numerous variants in an infected patient. These variants with an undiminished virulence can multiply rapidly in order to progress to AIDS. One of the targets to intervene in HIV-1 replication is the *trans*-activator of transcription (Tat), a major regulatory protein that transactivates the long terminal repeat promoter through its interaction with *trans*-activation response (TAR) RNA. In this study, HIV-1 infected patients (*n* = 120) from North India revealed Ser46Phe (20%) and Ser61Arg (2%) mutations in the Tat variants with a strong interaction toward TAR leading to enhanced transactivation activities. Molecular dynamics simulation data verified that the variants with this mutation had a higher binding affinity for TAR than both the wild-type Tat and other variants that lacked Ser46Phe and Ser61Arg. Other mutations in Tat conferred varying affinities for TAR interaction leading to differential transactivation abilities. This is the first report from North India with a clinical validation of CD4 counts to demonstrate the influence of Tat genetic variations affecting the stability of Tat and its interaction with TAR. This study highlights the co-evolution pattern of Tat and predominant nucleotides for Tat activity, facilitating the identification of genetic determinants for the attenuation of viral gene expression.

## Introduction

Human immunodeficiency virus type 1 (HIV-1) overcomes the host immune defense by rapid evolution and genetic diversification (Dougherty and Temin, 1988; Jetzt et al., 2000). Rapid replication without the benefit of proof-reading leads to the generation of a large number of mutations and recombination events (Wolinsky et al., 1996; Jetzt et al., 2000). This vigorous recombination allows HIV-1 to produce multiple groups, subtypes, sub-subtypes and recombinants in an infected patient (Wolinsky et al., 1996; Buonaguro et al., 2007). Of these, only highly virulent variants and recombinants are likely to adapt and spread in a population (Ho et al., 1995; Buonaguro et al., 2007). It is known that genetic and functional changes in HIV-1 genes accomplished with an emergence of recombination events (Blackard et al., 2002), could affect the virulence of HIV-1 resulting in vulnerability to AIDS despite antiretroviral therapy (ART) (Geretti, 2006; Kirchhoff, 2009; Sharp and Hahn, 2011; Santoro and Perno, 2013).

Among HIV-1 proteins, *trans*-activator of transcription (Tat) plays an important role in mediating the viral transcription (Okamoto et al., 1996; Zhu et al., 1997). Tat is expressed during the early stages of infection, which is encoded by two exons (exons 1 and 2) in a multiple spliced mRNA (Arya et al., 1985). Tat strongly activates transcription (Muesing et al., 1987) from long terminal repeat (LTR) promoter through a strong interaction with *trans*-activation response element (TAR) sequence at the 5′ end of the LTR (+1 to +59) (Dingwall et al., 1989; Buonaguro et al., 1994). During the binding to TAR and host factors Cdk9 and cyclin T1, Tat alters the transcription complex, recruiting a positive transcription elongation complex (P-TEFb), an elongation factor composed of cyclin T1 (CycT1) and Cdk9 that phosphorylates the C-terminal domain of RNA polymerase II leading to the increased production of viral RNA (Zhou and Rana, 2002). Tat contributes to the pathogenesis of HIV-1 through its pivotal role in replication, T-cell apoptosis, co-receptor regulation, cytokine induction, and other viral activities in the host cells (Fulcher and Jans, 2003; Romani et al., 2010).

HIV-1 produces highly divergent variants within a single patient, thereby overcoming the host immune responses, antiretroviral restriction factors, and other selection mechanisms (Konings et al., 2006; Coffin and Swanstrom, 2013). In India, the dominant HIV-1 type is subtype C (>95%), with the emergence of recombinants like A/C, A/E, and B/C (<2%) (Neogi et al., 2011; Ronsard et al., 2015), indicating that HIV-1 is under the stress toward the positive selection in the Indian population. Therefore, it is important to understand the nature of HIV-1 evolution in the population. Previous data showed that the genetic variations in Tat could lead to varying levels of LTR-driven transcription (Ronsard et al., 2014); however, how Tat variants differ in their transactivation abilities have not been explored. Tat is also known for its interaction with various cellular proteins and for its effect on modulating the viral gene expression, which in turn enhances the virulence (Bucci, 2015; Mediouni et al., 2015; Yuan et al., 2015) indicating one of the suitable targets against HIV-1 infection (Hamy et al., 2000; Burton et al., 2004; Nunnari et al., 2008; Sun et al., 2012; Ensoli et al., 2014). We earlier reported that Tat is a highly conserved protein with only a few genetic variations in the functional domains. However, a novel Ser46Phe mutation is prevalent among North Indian population (Ronsard et al., 2014). More than 20% of HIV-1 infected patients carry this mutation along with other mutations; therefore it is important to understand the role of these variants from the population.

Here, we report the differential ability of Tat variants with Ser46Phe mutation to significantly enhanced LTR transactivation (*P* < 0.05) compared to wild-type and other Tat variants that lack this mutation. We observed that variants with this mutation exhibited a strong interaction with TAR by *in vitro* and *in silico* studies, whereas other Tat variants exhibited varying levels of Tat–TAR mediated transactivation. Molecular dynamics (MDs) simulation data confirmed that Ser46Phe mutation exhibits a strong binding with TAR. Here, we illustrate how HIV-1 virus has evolved during selection pressure in the North Indian population with various mutations to adapt and survive in the host cells by enhancing its functional activity.

## Results

### Selection of Tat Variants for Functional Characterization

**FIGURE 1:**
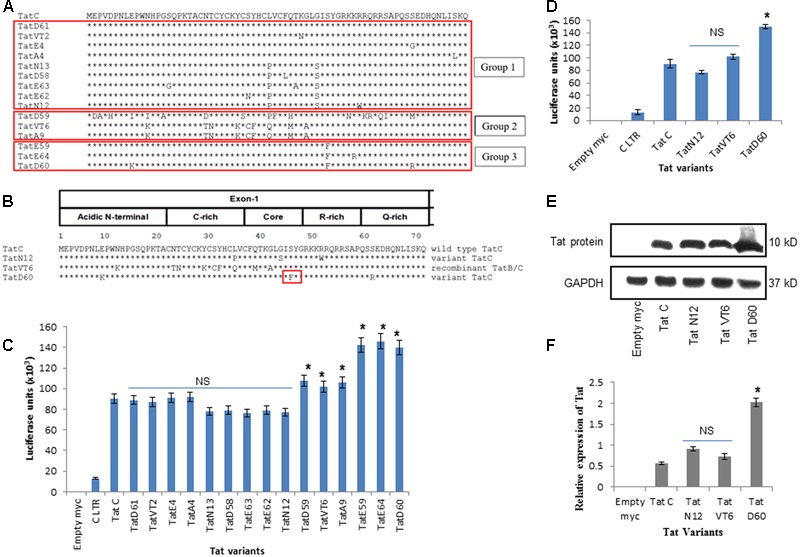
**HIV-1 LTR transactivation by Tat variants. (A)** Tat variants from HIV-1 infected individuals (*n* = 15) were aligned with wild-type Tat C (C.IN.93.93IN905). **(B)** Representative data of Tat variants[TatN12 (lack Ser46Phe) with Leu35Pro and Gly44Ser, TatVT6 (lack Ser46Phe) with B/C recombination and TatD60 (with Ser46Phe)] were aligned with wild-type Tat C. **(C)** HEK293 cells were co-transfected with pCMV-myc Tat variants and pGL3-Luc subtype C LTR. After 24 h of transfection, cells were harvested and lysed and luciferase activity was measured. The relative transactivation was expressed as mean luciferase units. Wild-type TatC was used as a reference Tat for comparison. Empty myc vector was used as a control. Subtype C LTR was used as a loading control. Luciferase activity of 15 Tat variants and wild-type Tat C normalized to empty pCMV-myc vector. **(D)** Luciferase activity of unique Tat variants and wild-type TatC normalized to empty pCMV-myc vector. Expression of Tat variants in HEK293 cells. HEK293 cells were transfected with pCMV-myc Tat variants and wild-type TatC. After 24 h, cells were harvested and immunoblotted with anti-myc antibody. The relative Tat protein expression was measured using ImageJ software. Wild-type TatC was used as a reference Tat for comparison. Empty myc vector was used as a control. GAPDH was used as a loading control. **(E)** Relative protein expression of Tat variants and wild-type TatC. **(F)** Quantification of Tat protein expression normalized to GAPDH. Error bar represents the standard deviation in triplicates. Statistical comparison of each Tat variant to Tat C was calculated by one-way ANOVA with the Tukey’s test (^∗^ denotes *P* < 0.05 and NS denotes not significant).

HIV-1 specimens from 120 patients were collected, and the Tat gene was amplified by polymerase chain reaction (PCR) and sequenced as described in Section “Materials and Methods.” From 120 variants, 15 variants were chosen based on mutations in the Tat gene and these variants were segregated into three groups (selected at least 3 variants in each group from total 120 variants which consisted of similar nucleotide changes) for LTR transactivation study (**Figure 1A**). Next, three Tat variants (TatN12, TatD60, and TatVT6) were selected based on their similarity in inducing LTR transactivation and carrying similar mutations (similar pattern of nucleotide changes) in the Tat gene (selected a variant from each group as a representative variant) for the TAR RNA interaction study which included TatN12, a subtype C variant (that lacked Ser46Phe) with Leu35Pro and Gly44Ser; TatD60, also a subtype C variant (with Ser46Phe) with Glu9Lys and Ser61Arg; and TatVT6, a B/C recombinant (that lacked Ser46Phe) (**Figure 1B**). Three groups were chosen based on the similarities in their genetic and functional activities of Tat (variants with similar nucleotide changes resulting in similar levels of LTR transactivation). It is worth mentioning that Ser46Phe mutation is also reported from neighboring countries like Myanmar and China (HIV database)^1^; however, the functional implication of this mutation on Tat–TAR mediated transactivation has not been studied. The phylogenetic tree was constructed with Tat variants to explain the Tat genetic variations occurring in the North Indian population (Supplementary Figure S1). Notably, the three variants (TatD60, TatE59, and TatE64 were used for transactivation study) and other variants (TatVT1, TatVT3, TatA7, TatN14, TatN17, TatCSW1, TatS5, TatS6) with Ser46Phe were clustered together in the phylogenetic tree indicating the proper classification of Tat variants into three different groups for the functional characterization. Further, the recombination event was confirmed in the Tat variants (TatVT6) using RIP (Recombinant Identification Program) with a confidence threshold 90% and a window size of 100 (Supplementary Figure S2).

### Role of Tat Mutations on Viral Transcription

To determine the transactivation activity of Tat variants, a luciferase assay was performed in HEK293 cells with 15 selected Tat variants of three groups. Wild-type TatC was used as a reference for baseline transactivation levels of Tat variants. Tat variants with similar nucleotide changes from all three groups (*n* = 15) showed similar levels of LTR transactivation suggesting that Tat-induced transactivation is dependent on the genetic determinants of Tat variants (**Figure 1C**). We observed that the levels of transactivation induced by TatN12 were similar or slightly lower than the wild-type TatC but not significantly; TatVT6-induced LTR transactivation was a slightly higher than wild-type TatC (*P* < 0.05). TatD60 carrying Ser46Phe showed a significantly higher level of transactivation (*P* < 0.05) than wild-type Tat C as well as other variants. In summary, the order of transactivation induced by variants TatD60 > TatV6 > TatN12 (**Figure 1D**) indicating Ser46Phe mutation play a significant role in transactivation, whereas, other natural mutations did not have a significant role on LTR transactivation. Similar pattern of transactivation was observed with the Tat variants along with the subtype B LTR (Supplementary Figure S3).

### Tat Mutations Affect Tat Protein Expression

To examine the effect of Tat variants on intracellular protein expression, Tat variants (TatN12, TatVT6, and TatD60) and wild-type Tat C were cloned into a pCMV-myc vector and were expressed and detected by immunoblotting with anti-myc antibody. Wild-type Tat C was used as a reference for comparison of protein expression. TatN12 and TatVT6 were expressed at similar levels of protein expression when compared to wild-type Tat C; while TatD60 (Ser46Phe) resulted in an elevated level of expression (**Figures 1E,F**) suggesting that Ser46Phe and Ser61Arg mutations in TatD60 could affect Tat protein expression.

### Tat Mutations Alter TAR Interaction

**FIGURE 2:**
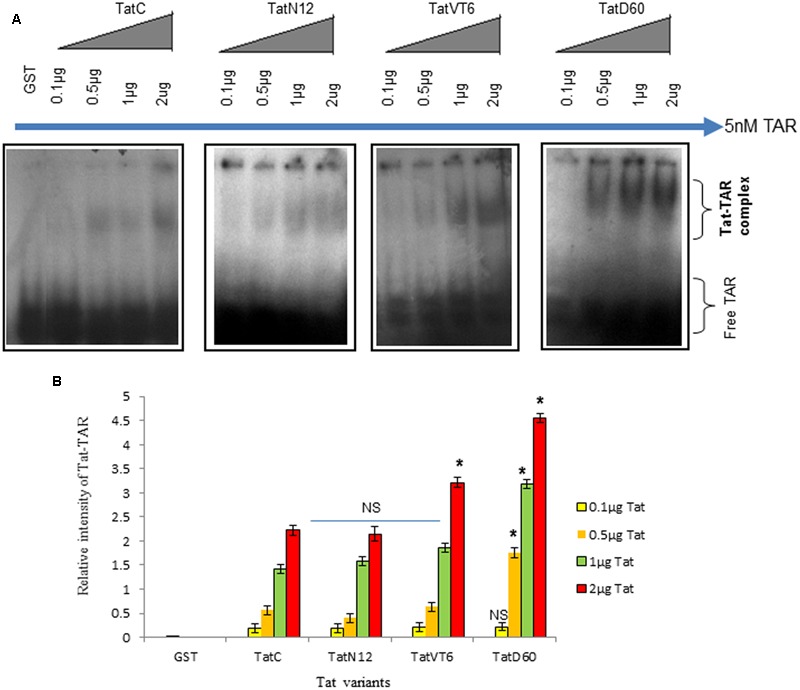
**Tat–TAR interaction by EMSA.** Tat variants and wild-type Tat cloned in pGEX-4T2 vector were expressed in BL21 (DE3) pLysS cells. Expressed Tat proteins were isolated and verified by immunoblotting with anti-Tat antibody. Increasing concentrations of purified Tat proteins were subjected to interact with subtype B TAR and these Tat ± TAR complexes were detected by autoradiography. The relative intensity of Tat variants–TAR complexes were measured using ImageJ software. Wild-type TatC–TAR complex was used as a reference Tat for comparison. GST was used as a control. Free TAR was used as a loading control. **(A)** Relative intensity of Tat variants–TAR complexes and wild-type TatC–TAR complex. **(B)** Quantification of Tat variants–TAR complexes normalized to empty vector. Error bar represents the standard deviation in triplicates. Statistical comparison of each Tat variant to Tat C was calculated by one-way ANOVA with the Tukey’s test (^∗^ denotes *P* < 0.05 and NS denotes not significant).

To examine the ability of Tat variants to interact with TAR, Tat variants were over-expressed and purified from *Escherichia coli* BL21 strain (Supplementary Figure S4). These proteins were incubated with TAR (synthesized by *in vitro* transcription) and Tat–TAR binding affinity was measured by Non-denaturing PAGE. Wild-type TatC–TAR complex formation was used as a reference. The binding affinity of TatN12 to TAR was less than wild-type TatC–TAR complex but not significantly; whereas the TatD60 variant carrying Ser46Phe showed significantly higher (*P* < 0.05) binding affinity with TAR RNA (**Figures 2A,B**). The binding affinity of the TatVT6–TAR complex was slightly higher than wild-type TatC–TAR complex but did not reach statistical significance.

### Tat Genetic Variations Determine the Protein Stability

**FIGURE 3:**
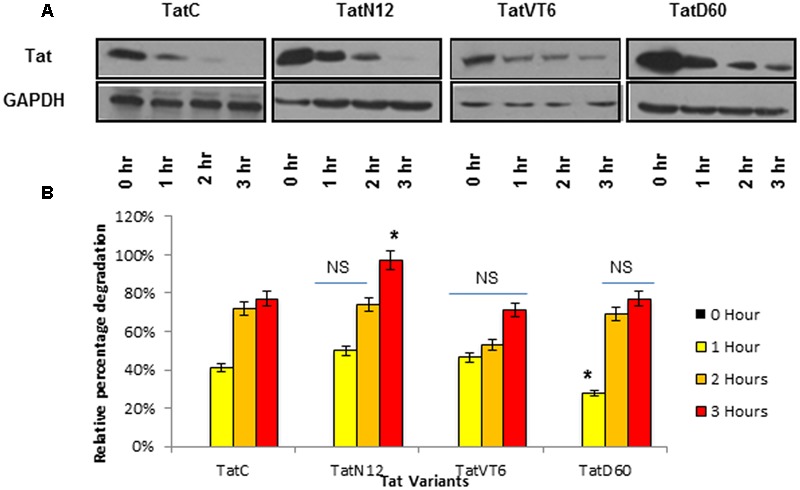
**Intracellular stability of Tat variants.** HEK293 cells were transfected with pCMV-Myc Tat variants and wild-type TatC, cycloheximide (100 μg/ml) was added after 24 h and cells were harvested at different time intervals and immunoblotted with anti-Tat antibody. The relative percentage of Tat protein degradation was measured using ImageJ software. Wild-type TatC was used as a reference Tat for comparison. Tat proteins expressed after 24 h of transfection (before adding cycloheximide) were used as controls. GAPDH was used as a loading control. **(A)** Relative protein expression of Tat variants and wild-type TatC at different time intervals. **(B)** Quantification of Tat protein degradation normalized to GAPDH. Error bar represents the standard deviation in triplicates. Statistical comparison of each Tat variant to Tat C was calculated by one-way ANOVA with the Tukey’s test (^∗^ denotes *P* < 0.05 and NS denotes not significant).

To determine whether Ser46Phe and other mutations play a role on Tat protein stability, a cycloheximide chase assay was performed. Briefly, HEK293 cells were transfected with Tat variants and incubated with cycloheximide for different time intervals, and the amount of Tat in cell lysates was quantified by western blotting. Wild-type Tat C protein stability was treated as baseline. After 3 h of cycloheximide treatment, the level of TatN12 was found to be reduced. In contrast, the protein levels of TatD60 and TatVT6 were detectable even after 3 h of treatment (**Figures 3A,B**) indicating that Ser46Phe and recombination events in TatD60 and TatVT6 respectively could possibly stabilize Tat protein.

### Influence of Genetic Variability on Tat Ubiquitination

**FIGURE 4:**
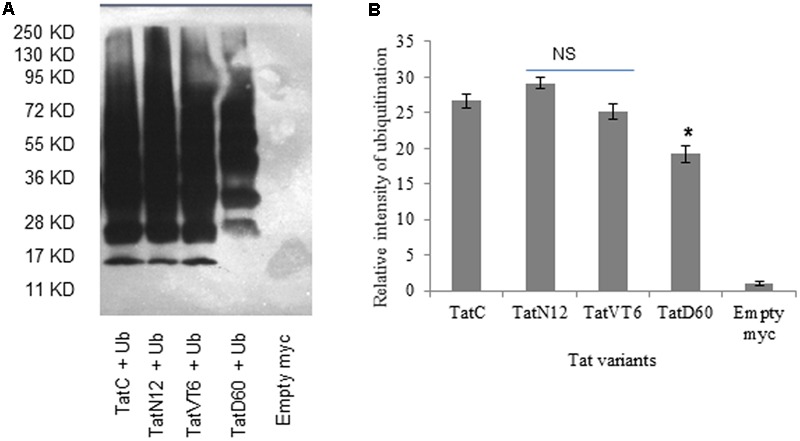
**Ubiquitination of Tat variants.** HEK293 cells were co-transfected with His6-Ubiquitin protein, and pCMV-myc Tat variants and wild-type TatC. After 24 h, cells were treated with MG132 (25 μM) for 8 h. Cell lysates were incubated with Ni-NTA beads and immunoblotted with anti-myc antibody. The relative intensity of Tat protein ubiquitination was measured using ImageJ software. Wild-type TatC was used as a reference Tat for comparison. Empty vector was used as a control. **(A)** Relative intensity of Tat proteins and wild-type Tat protein ubiquitination. **(B)** Quantification of Tat ubiquitination normalized to empty myc vector. Error bar represents the standard deviation in triplicates. Statistical comparison of each Tat variant to Tat C was calculated by one-way ANOVA with the Tukey’s test (^∗^ denotes *P* < 0.05 and NS denotes not significant).

To understand whether ubiquitination determines the differential stability of Tat proteins, we measured the level of ubiquitination of Tat variants in HEK293 cells. Wild-type Tat C ubiquitination was treated as baseline. TatN12 resulted in slightly higher or similar level of ubiquitination compared to wild-type Tat indicating less stability of this protein than TatD60 and TatVT6 proteins. TatVT6 showed a slightly lower ubiquitination than wild-type TatC while TatD60 showed less ubiquitination indicating that Ser46Phe and Ser61Arg could stabilize Tat protein (**Figures 4A,B**), though it is not known if Ser46 and Ser61 are targets for ubiquitination (Wang et al., 2007). The differential levels of ubiquitination of Tat variants appear to be dependent on the intracellular level of Tat protein expression.

### Tat Mutations Govern Stable Interaction with TAR

Next, we performed MD simulation to estimate the stability of TatD60–TAR and TatC–TAR complexes. We have made homology models of wild-type Tat and TatD60, and then docked with TAR RNA. TatC–TAR complex was treated as a baseline stability of the complex. MD simulation recorded the trajectories of Tat protein backbones and TAR in the docked complex for 20 nanoseconds (ns) in an aqueous environment. Root mean square deviation (RMSD) of Tat protein backbone and TAR ribonucleotides at *t* = 0 ns and *t* = 20 ns were compared to predict the stability of the complexes.

**FIGURE 5:**
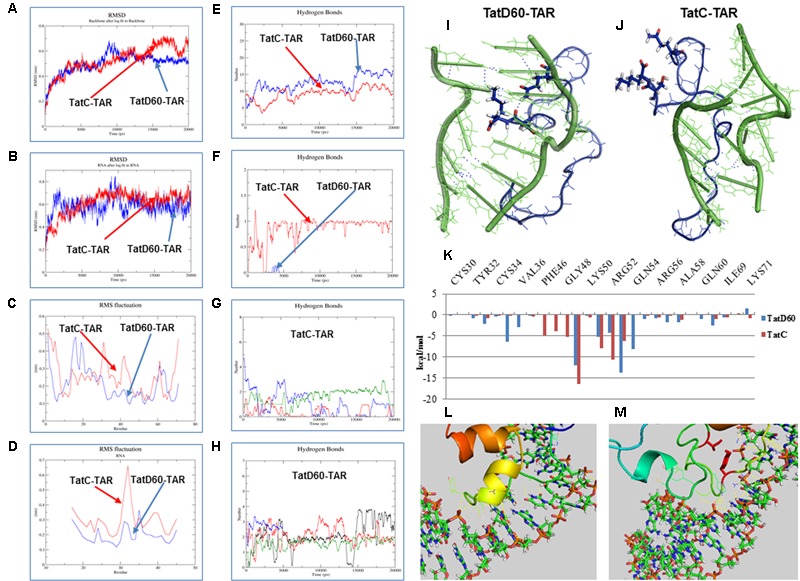
**Molecular dynamic (MD) simulation of Tat protein with TAR.** MD simulation for Tat–TAR was carried out for 20 ns to predict root mean square deviation (RMSD) of Tat variants. **(A)** RMSD of Tat residues in wild-type TatC–TAR (Red line) and TatD60–TAR (Blue line). **(B)** RMSD of TAR ribonucleotides in wild-type TatC–TAR (Red line) and TatD60–TAR (Blue line). **(C)** Root mean square fluctuation (RMSF) of Tat individual residues in wild-type TatC–TAR (red line) and TatD60–TAR (blue line). **(D)** RMSF of TAR individual ribonucleotides in wild-type TatC–TAR (red line) and TatD60–TAR (blue line). Hydrogen bonds in Tat–TAR complex. Number of H-bonds formed between Tat variants and TAR per time frame was measured; **(E)** number of H-bonds in wild-type TatC–TAR complex (red line) and TatD60–TAR complex (blue line). **(F)** Number of H-bonds formed at Ser46th position in wild-type TatC–TAR complex (red line) and at Phe46 in TatD60–TAR complex (blue line). **(G)** Number of H-bonds formed by TAR ribonucleotides of A22 (black line), U23 (red line), C24 (green line), and U25 (blue line) in wild-type TatC–TAR complex. **(H)** Number of H-bonds formed by TAR ribonucleotides of A22 (black line), U23 (red line), C24 (green line) and U25 (blue line) in TatD60–TAR complex. Tat variants interaction with TAR during simulation. Tat protein (blue) and TAR (green) interaction in the bulge region at the end of 20 ns simulation was captured. H-bonds represented in dotted blue lines. **(I)** Structure of wild-type TatC–TAR complex during 20 ns simulation. **(J)** Structure of TatD60–TAR complex during 20 ns simulation. Binding free energy of Tat–TAR complex. **(K)** Relative residue-wise energy contribution for binding wild-type TatC–TAR and TatD60–TAR complexes. Only selected residues with important contribution are shown. Comparison of crystal structure and modeled structure of Tat–TAR complex. Comparison of docked structure of Tat–TAR complex with homology modeled Tat structure and with the crystal structure (PDB ID: 5L1Z) of Tat. **(L)** Crystal structure complex of TatD60–TAR. **(M)** Homology modeled structure complex of TatD60–TAR.

Root mean square deviation of TatD60 backbone was observed at around 5 Å and remained stable throughout the simulation, except for a brief period of higher shift between 8 and 10 ns (**Figure 5A**). In contrast, RMSD for TatC backbone showed more fluctuations. In particular, the upward movement observed after 8 ns was not completely stabilized at the end of the simulation. The average length of protein backbone RMSD for the final 5 ns of simulation, 5.2 Å for TatD60 and 5.7 Å for wild-type TatC, indicated that TatD60–TAR complex had higher stability at equilibrium than wild-type TatC–TAR complex.

Root mean square deviation of TAR ribonucleotides in TatD60–TAR complex showed more drift when compared to TatC–TAR complex (**Figure 5B**), particularly from 8 to 10 ns. The comparison of extracted TAR structure alone at different time frames showed that RMSD was reduced by 19.3 Å in TatD60–TAR complex and by 7.8 Å in wild-type TatC–TAR complex. This reduction could be due to Ser46Phe mutation in TatD60 which created a conformational change in order to bind strongly with TAR ribonucleotides and produced a more stable TatD60–TAR complex.

Next, root mean square fluctuation (RMSF) of each amino acid in Tat proteins differed between TatD60 and TatC. For TatC, maximum fluctuations were observed for five residues at Ser31, Tyr32, His33, Lys41, and Gly42 in addition to the N-terminal and the C-terminal residues (**Figure 5C**). For TatD60, maximum fluctuations were observed for only three residues at Gly15, Ser16, and Lys19 in addition to the N-terminal residues (**Figure 5C**). RMSF was lower for Ser70 and Lys71 in TatD60 when compared to wild-type TatC.

In the case of TAR, ribonucleotides in the bulge region (U23, C24, and U25) involved in Tat interaction, have similar RMSF when interacting with TatC or TatD60, however the loop ribonucleotide G32 appeared to be more flexible in TatC–TAR complex (**Figure 5D**). RMSF of each TAR ribonucleotides in TatD60–TAR complex showed less drift when compared to TatC–TAR complex (**Figure 5D**).

### Hydrogen Bonds in Tat–TAR Complexes

In wild-type TatC, Ser46 resulted in a single H-bond with TAR (occupancy ∼71%) (**Figure 5E**), while the corresponding position was Phe46 in TatD60 resulting in one H-bond with TAR only transiently (occupancy below 1%); evidently, the single Ser46Phe mutation had a considerable effect on the H-bond profile of the TatD60–TAR complex (**Figure 5F**). In case of ribonucleotides in TAR, the wild-type Tat had less interaction (**Figure 5G**) than compared to TatD60 (**Figure 5H**) indicating strong H-interaction of TatD60 toward TAR.

Further, analysis of simulated structures at 20 ns revealed potentially critical differences in Tat–TAR interaction at bulge ribonucleotides A22, U23, C24, and U25. TatD60–TAR complex showed stacking interaction between U23 and A22, and standard base-pairing between coplanar bases A22 and U40 (**Figure 5I**) whereas both these features were missing in the TatC–TAR complex (**Figure 5J**). Overall, the number of hydrogen bonds formed between Tat variants and TAR represented in the order of TatD60 > TatVT6 > TatN12.

### Binding Free Energy and its Residue Wise Decomposition Analysis of Tat–TAR Interaction

To understand the changes in free energy during formation of Tat variants–TAR, complexes were computed using residue-wise energy decomposition analysis by MM/GBSA method. TatC–TAR was used as a reference for Tat–TAR complex free energy decomposition. ΔG_bind_ of TatD60–TAR was estimated to be -80.49 kcal/mol, whereas in TatC–TAR ΔG_bind_ was -67.14 kcal/mol (Supplementary Table S1). The main forces contributing to the difference between ΔG_bind_ for TatD60–TAR and TatC–TAR were van der Waals and electrostatic interactions.

Residue-wise binding energy decomposition (**Figure 5K** and Supplementary Table S2) showed significant contributions by the following residues in TatD60: Leu35 (-6.447 ± 9.452), Arg49 (-12.070 ± 15.687), Lys51 (-5.266 ± 18.739), Arg53 (-13.688 ± 12.174), Gln54 (-8.058 ± 9.557), and Asn67 (-8.708 ± 6.229), and the following residues in TatC: Arg49 (-16.524 ± 17.477), Lys51 (-7.876 ± 14.652), Arg52 (-10.619 ± 16.843), and Arg53 (-6.192 ± 24.679). In contrast, both TatC and TatD60, Gln54 (0.065 ± 7.828) and Asn67 (0.181 ± 7.948) disfavored the binding ability. These findings further support evidence for a higher affinity of TatD60 for TAR.

### Comparison of Crystal Structure and Modeled Structure of Tat–TAR Complex

Further, we have analyzed the Tat crystal structure (PDB ID: 5L1Z) with TAR RNA (**Figure 5L**) and then the complex was compared to the Tat homology modeled complex with TAR RNA (**Figure 5M**). In spite of having few differences in the Tat modeled protein and in the Tat crystal structure, their binding pattern with TAR RNA were found to be similar and also both the complexes were following the same trend in making the hydrogen bonds. The residues Lys50, Lys 51, Arg52, Arg55, Ser57, Asn67, and Ser70 are the major residues contributing more toward the Tat and TAR binding were the same in corresponding residues in the crystal structure (PDB ID: 5L1Z). The additional hydrogen bonds formed in the crystal structure were Thr40 and Tyr46. Those additional hydrogen bonds with hydrogen bond distance 2.5 and 2.8 Å were distinct from the Tat modeled structure as it was modeled as extended loop and it was away from the C19 and U22 respectively.

### Clinical Status of HIV-1 Infected Patients with Tat Variants

In this study, CD4 counts were follow-up for 6 months for HIV-1 infected patients (*n* = 15) which included: group 1 was consisted of HIV-1 infected patients (*n* = 9) lacking Ser46Phe in Tat C; group 2 was consisted of HIV-1 infected patients (*n* = 3) lacking Ser46Phe in Tat B/C recombinant; and group 3 was consisted of HIV-1 infected patients (*n* = 3) with Ser46Phe and Ser61Arg in Tat C.

Of these 15 patients, the mean CD4 count in group 1 patients (*n* = 9) was 470, and after 6 months of ART, the mean value of CD4 count increased to 557 with ±87 standard deviation of the mean value. This showed that the patients in group 1 benefited from ART and indicated that there was no effect of Tat mutations on CD4 counts. However, two patients (TatE4 and TatD58) had almost constant CD4 counts while the other seven patients had increased CD4 counts. Next, the mean CD4 count in group 2 patients (*n* = 3) was 613, and after 6 months of ART, the mean value of CD4 count decreased to 578 with ±35 standard deviation of the mean value. This showed that the CD4 counts of the patients in group 2 could be affected by B/C Tat; however, the changes were not significant. In group 3 patients (*n* = 3), the mean CD4 count was 493. After 6 months of ART, the mean value of CD4 count decreased to 359 with ±134 standard deviation of the mean value. This showed that the patients in this mutation group 3 could be affected due to Ser46Phe and other mutations in this group (Supplementary Table S3).

In the total of 15 patients, the mean viral load after ART in group 1 patients (*n* = 9) was less than 50 copies/ml. This showed that the patients did not affected by the transactivation level induced by Tat mutations in this group (particularly TatN12 is <50 copies/ml). However, two patients (TatE4 and TatD61) had slightly detectable viral loads but not significantly. Next, the mean viral load in group 2 patients (*n* = 3) was slightly increased to 158 copies/ml. This showed that the viral load of the patients could be affected by the transactivation level induced by B/C Tat in this group (particularly TatVT6 is 182 copies/ml). In group 3 patients (*n* = 3), the mean viral loads was 265 copies/ml indicating that the patients in this mutation group 3 could be affected by the transactivation level caused due to Ser46Phe and other mutations in this group (particularly TatD60 is 346 copies/ml), however, there could be also multiple other factors might affect both the CD4 count and the viral load indicating the need for the further study in a large sample size (Supplementary Table S3).

## Discussion

A rate-limiting factor in the management of HIV infections, is the plethora of genetic variations leading to failure of clinical trials (Santoro and Perno, 2013). Each geographical region has its own profile of HIV-1 types, subtypes, recombinants and mutations, and these genetic variations lead to differential potential in inducing HIV-1 pathogenesis (Rodriguez et al., 2009). We previously reported the differential expression of certain viral genes of subtypes B and C with respect to their functions (Sood et al., 2008; Gupta and Banerjea, 2009). The viral or cellular components responsible for these differences have not been studied in details and in most cases, the molecular details of these genetic determinants have not been well-explored. Our previous reports showed that the functional role of viral proteins is mediated by the genetic determinants present in the viral genes (Siu et al., 2013; Zhu et al., 2013). It is known that Tat subtype-specific variations exhibit widely differing viral activities including their ability to activate the HIV-1 LTR promoter (Li et al., 2012); however, the functional and clinical consequences of the substantial genetic variations of Tat occurring in North India have not been studied. The growing number of studies on Tat-based inhibitors against HIV-1 replication (Hamy et al., 1997; Lalonde et al., 2011) indicates the importance of this study.

In this regard, we attempted to understand the differential ability of Tat variants to activate LTR transactivation through their ability to interact with TAR using *in vitro* and *in silica* approaches. Tat protein is highly conserved protein among North Indians. For viral replication, it is important that Tat retains its functional activity because any changes in the genetic makeup of Tat could lead to drastic modulation in transcription-coupled pathogenicity. In our survey of 120 Tat sequences from 120 HIV-1 infected patients, we found certain point mutants and B/C recombinants commonly occurring among North Indians. In order to understand the role of these natural mutations, we carried out genetic and functional analyses in correlation with clinical CD4 counts. Both *in silico* and *in vitro* studies show that the Ser46Phe mutation in Tat results in enhanced transactivation. Next, to demonstrate how Ser46Phe contributed toward high transactivation activity. Wild-type TatC (that lacked Ser46Phe) was used as a reference Tat to compare their differential abilities to interact with TAR and their intracellular protein stability. We also used two other subtype C variants, TatN12 and TatVT6 (that lacked Ser46Phe) found in the studied population. These variants showed less transactivation than TatD60 (with Ser46Phe).

Tat transactivates HIV-1 LTR by binding to TAR, which is a critical step in the process of transcription (Huq et al., 1997; Rana and Jeang, 1999). Residues vital for Tat function include Lys28, Lys41, Lys50, Lys51, and Lys71 (for acetylation); Arg57 and Arg56 (for TAR interaction); and Tyr47, Cys22, Cys31, and Cys34 (for LTR transactivation) (Huo et al., 2011). Most of these residues were highly conserved among North Indians; however, we found that variants with Ser46Phe showed higher transactivation of LTR than TatC indicating the importance of this mutation. We studied 15 Tat variants for their ability to induce transactivation and found that variants with similar mutations resulted in similar levels of transactivation indicating the vital role of genetic variations in modulating functions. From 15 variants, we selected three variants namely TatN12 (Leu35Pro; Gly44Ser), TatD60 (Ser46Phe), and TatVT6 (B/C recombinant) as representative variants to understand the role of genetic determinants on transactivation, TAR interaction and stability. TatVT6 showed increased transactivation, which may be due to subtype C specific changes in the N-terminus and subtype B specific changes in the C-terminus. TatN12 showed less transactivation (not significant) than wild-type TatC which could possibly be due to unique mutations in TatN12 leading to weak interaction with TAR. However, the biological and clinical relevance of the reported Tat mutations remains to be established with reference to TAR sequence variations from patients.

MD simulation is a useful technique to determine the stability of biological complexes (Hornak et al., 2006; Deng et al., 2011; Johnson et al., 2012; Venken et al., 2012; Zhao et al., 2013; Vijayan et al., 2014), here, we utilized this technique to calculate the fluctuations and the binding free energy of Tat–TAR complex (Nifosi et al., 2000). Data generated from MD simulation substantiate our *in vitro* studies and provide further insights into a stronger interaction of TatD60 with TAR. The TatD60–TAR complex was more stable during the simulation with a stronger interaction between Tat and TAR. As predicted in the electrophoretic mobility shift assay (EMSA) experiment, the interaction between TatD60 (with Ser46Phe) and TAR were different in the simulation experiment. This difference has led to additional H-bond interactions between the residues namely Tyr26, Tyr29, Cys30, Ser31, Tyr47, and Ser70 in TatD60 to TAR interaction, whereas wild-type TatC lacks these residue interactions toward TAR. In case of TAR, ribonucleotide U23 and A22 led to additional H-bonds interaction with TatD60, resulting in strong TAR interactions. Further, binding free energy calculation and residue-wise energy decomposition analyses clearly suggested that TatD60–TAR complex interacted more stably when compared to wild-type TatC–TAR complex. Particularly, the residues Gln54 and Asn67 in TatD60, but not in TatC, contributed to the binding.

TatD60 appeared to have a more stable half-life than other TatC variants within the host cells, possibly due to lower level of ubiquitination, it is clear from the fact that it has enhanced transactivation. Given its enhanced potency for transactivation and its higher stability in the cells, TatD60 has the potential to be more virulent than other TatC variants studied here. This hypothesis was supported by our observation of CD4 counts in the patients that were the source of these variants (Supplementary Table S3). However, these predictions need to be tested in a large sample population. Correlating Tat variants, especially variants with Ser46Phe, with the course of the disease, as well as response to ART, would provide molecular and clinical insights to managing HIV-1 patients in North India.

There could be other factors involved in the decline of CD4 counts in HIV-1 infected patients, however, we believe that this study with the control groups (that lacked Ser46Phe) showcase the genetic variations of Tat that could be one of the reasons for the decline. At present, it was difficult to draw a conclusion on CD4 counts and viral loads with the small sample size and warrant further need for monitoring the genetic evolution of HIV-1 strains among North Indians.

Future studies to evaluate the binding efficiency of Tat variants during complex with P-TEFb (Schulze-Gahmen et al., 2014) should further support this relationship. Taken together, this study illustrates the importance of point mutations for modulating the specific functional activities of Tat, which include increasing transactivation levels. These variants have the ability to emerge as a virulent HIV-1 strains in North India. Retrospective studies on Tat–TAR complex show that inhibition of this complex is an attractive target for developing novel antiviral drugs (Yang, 2005; Mousseau et al., 2015) and Tat could be also used as a vaccine candidate (Ensoli et al., 2016); therefore, manipulation of Tat–TAR interaction by silencing important residues is critical in forming the Tat–TAR complex as this would provide a strategy for suppressing viral gene expression. Continued studies are needed to elucidate how Tat variants manipulate the host immune cells in this population. Further, our results suggest that the nucleotides of Tat and their functions should be routinely investigated as a correlation of transactivation levels. Thus, this study provides valuable insights into the evolving events underlying the ability of the virus to adapt and enhance its replication by generating mutations and recombination events.

## Materials and Methods

### Ethics Statement and Collection of Samples

This study design was approved by Research Project Advisory Committee, Institutional Biosafety Committee, and Institutional Ethical Committee for Human Research of University College of Medical Sciences (UCMS) and Guru Teg Bahadur (GTB) Hospital, Delhi, India, and from Post Graduate Institute of Medical Education and Research (PGIMER), Chandigarh, India. These institutes are mentored by the National AIDS Control Organization (NACO), Ministry of Health and Family Welfare, Government of India that provides free ART to HIV-1 seropositive patients under a structured HIV/AIDS Control Program. Written informed consent was obtained from HIV-1 infected adult patients (*n* = 105) and from the guardians of HIV-1 infected children participants (*n* = 15) in this study. Blood samples were collected from HIV-1 infected patients (*n* = 120; males = 68, females = 52) registered and monitored at immunodeficiency clinics in GTB Hospital and PGIMER during the period from 2004 to 2010.

### Estimation of CD4 Counts and Viral Loads

HIV-1 infects CD4+ T cells and reduces these cells to a minimum level which is an indicator for the early risk of acquired immunodeficiency syndrome (AIDS) than viral load. People living with HIV AIDS (PLHA) patients under ART have shown improved CD4 counts and longer life span. ART was started in HIV-1 infected patients having CD4 count below 350/ml and children with varying counts. In this view, our study was undertaken to evaluate the correlation between Tat genetic variations on CD4 counts after 6 months on ART. From ART patients, blood was collected (CD4 counts were measured at this stage) and Tat gene was amplified, after 6 months on ART, once again CD4 counts and viral loads were measured and analyzed statistically. The CD4 count was estimated by flow cytometry using manufacturer’s directions (BD Biosciences) and viral load was measured using Real Time PCR (Taqman). The viral load of less than 50 copies/ml was defined as a viral suppression or undetectable viral load.

### DNA Isolation and Polymerase Chain Reaction (PCR)

Genomic DNA was extracted from PBMCs of HIV-1 infected patients by QIAamp DNA Blood Mini Kit (Qiagen) and HIV-1 subtype C (Indian isolate 93IN905 GenBank accession number AF067158 obtained from AIDS Research and Reference Reagent Program, Division of AIDS, NIAID, NIH) were used for amplification by PCR using the following primers:

**Table:** 

Forward primer:	5′-ATGGAGCCAGTAGATCCTAACCTA-3′
Reverse primer:	5′-TTGCTTTGATATAAGATTTTGATGA
	TCCT-3′

PCR was carried out in a 15 μl reaction volume. The reaction mixture contained 500 ng genomic DNA (2 μl), 10× PCR Buffer (1.5 μl), 10 mM dNTP mix (0.37 μl), 1 μL of each primer (25 pmol), 0.25 μl of Takara Taq DNA polymerase and 8.88 μl of DNase/RNase free water. PCR conditions for the above primer sets were as follow: initial denaturation at 94°C for 5 min (1 cycle), 30 cycles of denaturation at 94°C for 15 s, annealing at 63°C for 30 s and extension at 72°C for 40 s, and a final extension at 72°C for 5 min (1 cycle). PCR amplified products were analyzed on 1.5% agarose gel. Tat amplified from Indian HIV-1 subtype C (C.IN.93.93IN905) was used as wild-type TatC for comparison study with Tat variants. The Tat sequences were amplified both from the PBMCs viral DNA and also from the plasma viral RNA to find the exact genetic variations. The nucleotide sequences of Tat from both the source were similar in the pattern.

### Cloning, Sequencing, and HIV-1 Sub-typing

The gel purified PCR products were cloned into pGEM-T easy vector (Promega). The ligation reaction was incubated at 4°C for 10 h then the ligation mix was added to LB ampicillin plates with *E. coli* DH5α strain. The plates were incubated overnight at 37°C. The positive clones were selected by picking a single colony and grown in 5 ml LB Broth with ampicillin (100 μg/ml) and incubated overnight at 37°C. Plasmid DNA was isolated from the culture by QIAprep Spin Mini Kit (Qiagen). The positive clones were screened by restriction digestion of plasmid DNA with *EcoRI* in a 10 μl reaction volume at 37°C for 2 h. The digested products were analyzed on a 1.5% agarose gel. The positive clones were commercially sequenced from LabIndia and SciGenom laboratories. The nucleotide sequences were assembled and error was checked by using BLAST to search for sequence similarities to previously reported sequences in the databases and to eliminate potential laboratory errors. HIV-1 sub-typing, recombination, phylogenetic tree and mutational analyses have been carried out as described (Ronsard et al., 2014, 2015).

### Plasmids and Antibodies

TatC (lack Ser46Phe) and Tat variants (TatN12, TatD60, TatVT6) were cloned into: (a) mammalian expression vector pCMV-myc vector (Clonetech) under the CMV promoter for functional studies, and (b) prokaryotic expression vector pGEX-4T-2 (Invitrogen) to obtain GST-tagged proteins. HIV-1 subtype B TAR was cloned in pcDNA3.1 (Invitrogen) for TAR synthesis to determine Tat–TAR binding activity. Anti-Tat antibody (NIH AIDS Reagent Programme), Anti-myc antibody (Clontech), Anti-GAPDH antibody (Cell Signaling Technology), Anti-rabbit IgG conjugated to HRP (Jackson Immunoresearch), and Anti-Mouse IgG conjugated to HRP (Jackson Immunoresearch) were used in western blotting.

### Cell Culture and Transfection

Human embryonic kidney (HEK) 293 cells (NIH AIDS Reagent Programme) were maintained in Dulbecco’s modified Eagle’s medium (DMEM) (Himedia Laboratories) supplemented with L-glutamine and sodium pyruvate, fetal calf serum (10%), penicillin (100 U/ml), streptomycin (0.1 mg/ml), and amphotericin B (0.25 μg/ml) at 37°C in the presence of 5% CO_2_. Cells were transfected with lipofectamine 2000 (Invitrogen) in serum free DMEM media.

### Western Blotting

HEK293 cells were transfected with 1 μg of pCMV-myc Tat variants (TatN12, TatVT6, and TatD60) and wild-type TatC. After 24 h, cells were harvested and total protein was extracted using RIPA lysis buffer (Invitrogen). The amount of protein was estimated by BCA Assay (Pierce). Tat proteins were run on 12% SDS-PAGE and transferred to the nitrocellulose membrane (BIORAD) using standard methods (Chaudhuri et al., 2015; Mohankumar et al., 2015; Sridharan et al., 2015). The membrane was incubated with anti-myc antibody followed by Anti-rabbit IgG conjugated to HRP. The membrane was developed using ECL reagent (Amersham). GAPDH was used as a loading control and the expression of proteins was normalized with the amount of GAPDH. 1 μg of the empty pCMV-myc vector was used as a control in all the experiments and the experiment was repeated three times for confirmation of the result.

### Luciferase Reporter Assay

HEK293 cells were co-transfected with 200 ng of pCMV-myc Tat variants and wild-type TatC in each well of 6 well plate along with 50 ng of pGL3-Luc vector containing subtype C LTR. Cells were transfected only with subtype C. LTR construct was used as a control. After 24 h of transfection, cells were harvested and lysed with reporter lysis buffer (Promega) and luciferase activity was measured in the luminometer. 200 ng of the empty pCMV-myc vector was used as a control and the luciferase activity was normalized to the empty vector; the experiment was performed in triplicate.

### Purification of Tat Proteins

Tat variants and wild-type Tat were cloned into pGEX-4T-2 vector. *E. coli* BL21 (DE3) PlysS cells were transformed with recombinant plasmids and grown at 37°C overnight. Recombinant protein expression was induced by IPTG for 3 h at 37°C. Cells were harvested, disrupted and recombinant proteins were purified using Glutathione-agarose (Pierce) using manufacturer’s directions.

### Electrophoretic Mobility Shift Assay (EMSA)

Subtype B TAR was cloned between HindIII and BamHI site in pCDNA3 vector (Promega). ^32^P-labeled TAR was transcribed *in vitro* using T7 RNA polymerase. TAR was incubated with increasing amounts of purified Tat protein (0.1–2 μg) for 10 min on ice, followed by 10 min at 37°C with binding buffer (Promega). The reaction was stopped by adding 4X gel loading buffer and Tat variants with TAR complexes were analyzed on 4% Non-denaturing polyacrylamide gels and autoradiography was done. 1 μg of the empty pCMV-myc empty vector was used as a control and the expression of Tat–TAR binding was normalized with interaction with empty vector; the experiment was repeated three times.

### Cycloheximide Chase Assay

HEK293 cells were transfected with 1 μg of pCMV-myc Tat variants. After 24 h, cycloheximide was added (final concentration 100 μg/mL). Cells were harvested at different time intervals (0, 1, 2, and 3 h). Cell lysates were made with 1X RIPA lysis buffer and resolved by 12% SDS-PAGE. Anti-Tat antibody was used for detection by immunoblotting. GAPDH was used as a loading control and the expression of proteins was normalized with the amount of GAPDH. Tat proteins expressed after 24 h of transfection (before adding cycloheximide) were used as controls; the experiment was repeated three times.

### *In Vitro* Ubiquitination Assay

HEK293 cells were co-transfected with 1 μg of pCMV-myc Tat variants and 1 μg of His6-Ubiquitin Protein for 24 h and processed as previously described (Verma et al., 2011). The expression of proteins was normalized with the amount of empty vector. 1 μg of the empty pCMV-myc vector was used as a control; the experiment was repeated three times.

### Homology Models and MD Simulations

Homology models of Tat protein variants were generated using the solution structure of Tat protein as a template (PDB ID: 1TAC) and a crystal structure (PDB ID: 5L1Z) using Modeller 9v8 (Eswar et al., 2007) and then docked using HADDOCK web server (Guru Interface) (Eisenberg et al., 1997). Models were validated using PROCHECK (Laskowski et al., 1996) and the 3D-1D score of Verify3D (Bowie et al., 1991; Luthy et al., 1992). MD simulations were performed using GROMACS v4.5.6 (Van Der Spoel et al., 2005; Pronk et al., 2013) with AMBER99SB-ILDN force field (Lindorff-Larsen et al., 2010). Tat–TAR complex was solvated in a cubic box using TIP3P water model. The solvated systems were subjected to energy minimization using steepest descent and conjugate gradient algorithms keeping energy gradient convergence cut off of 10 kJ mol^-1^ nm^-1^. LINCS algorithm was used to calculate all the covalent bonds with hydrogen. The time step was kept at 2 femtoseconds (fs) for the simulation. The cut-off distance of 10 Å was used for all short-range non-bonded interactions and 12 Å Fourier grid spacing in PME was used for long-range electrostatics. NVT and NPT steps were run for 250 picoseconds (ps) and the final production run was done for 20 nanoseconds (ns).

### Binding Free Energy Calculations

The binding free energy of Tat–TAR complex was estimated by using MM/GBSA python scripts implemented in Amber11 package (Kollman et al., 2000; Campanera and Pouplana, 2010). Energy calculations were done over 5000 frames of 5 ns trajectory. Residue-wise energy decomposition studies were performed over the same trajectory using Amber decomposition script, which highlights important interactions between Tat proteins and TAR, and to identify the crucial residues in Tat proteins.

### Statistical Analysis

Data were analyzed using the SPSS 7.5-Windows student version software (SPSS, Inc., Chicago, IL, USA). One-way ANOVA followed by Tukey’s test was used to assess statistical significance between groups (*P* < 0.05 represents significance and *P* < 0.01 represents high significance) (Mohankumar et al., 2014a,b).

### Accession Numbers

Sequences of 120 Tat variants are available at – (GenBank: FJ432068-FJ432079, FJ210870-FJ210875, EU583126-EU583128, EU551665, FJ429357, FJ429358, HQ110624-HQ110630, HQ110608-HQ110623, JQ918787-JQ918788, GU451679-GU451681, and HQ011384-HQ011385). Sequences of unique Tat variants are available at (GenBank: TatN12 – HQ110625, TatVT6 – FJ432073, TatD60 – HQ110614).

## Author Contributions

LR and AB conceived and designed the experiments. LR and NG performed the experiments. VS performed simulation experiment. LR, VS, KM, TR, SS, DR, MC, and AB analyzed and interpreted the data. LR, NG, VS, KM, TR, SS, SP, BK, DR, SC, MC, VR, and AB contributed reagents/materials/analysis tools. LR, VS, TR, MC, VR, and AB wrote the manuscript. LR, KM, TR, BK, VR, and AB edited the manuscript.

## Conflict of Interest Statement

The authors declare that the research was conducted in the absence of any commercial or financial relationships that could be construed as a potential conflict of interest.

## Footnotes

1 http://www.hiv.lanl.gov/
