# Supplementary material for: Impact of Genetic Variations in HIV-1 Tat on LTR-Mediated Transcription via TAR RNA Interaction
Source: Front Microbiol. 2017 Apr 21;8:706. doi: 10.3389/fmicb.2017.00706 (PMC5399533; doi:10.3389/fmicb.2017.00706)
Supplement: Supplementary file 1 [file Data_Sheet_1.docx]

**Impact of genetic variations in HIV-1 Tat on LTR-mediated transcription via TAR RNA interaction**

Larance Ronsard^1,2*#^, Nilanjana Ganguli^1†^, Vivek Kumar Singh^3†^, Kumaravel Mohankumar^4†^, Tripti Rai^5†^, Subhashree Sridharan^4^, Sankar Pajaniradje^4^, Binod Kumar^6^, Devesh Rai^7^, Suhnrita Chaudhuri^8^, Mohane Selvaraj Coumar^3^, Vishnampettai G Ramachandran^2^ and Akhil C Banerjea^1*^

^1^Laboratory of Virology, National Institute of Immunology, New Delhi, India.

^2^Department of Microbiology, University College of Medical Sciences and Guru Teg Bahadur Hospital, Delhi, India.

^3^Centre for Bioinformatics, School of Life Sciences, Pondicherry University, Pondicherry, India.

^4^Department of Biochemistry and Molecular Biology, Pondicherry University, Pondicherry, India.

^5^Department of Gastroenterology and Human Nutrition, All India Institute of Medical Sciences, Delhi, India.

^6^Department of Microbiology and Immunology, Rosalind Franklin University of Medicine and Science, North Chicago, Illinois, USA.

^7^Department of Microbiology, All India Institute of Medical Sciences, Delhi, India.

^8^Department of Neurological Surgery, Northwestern University, Chicago, Illinois, USA.

#Present Address: Ragon Institute of MGH, MIT and Harvard, 400 Technology Square, Cambridge, MA 02139, USA

†Equally contributed in this study.

***Correspondence authors**

Dr. Akhil C. Banerjea, Chief Staff-Scientist VII, Laboratory of Virology, National Institute of Immunology, Aruna Asaf Ali Marg, New Delhi-110067, India; Off No: +91-011-26703616; Email IDs: [akhil@nii.res.in](mailto:akhil@nii.res.in), [akhil@nii.ac.in](mailto:akhil@nii.ac.in)

Dr. Larance Ronsard, Research Fellow, Ragon Institute of MGH, MIT and Harvard, 400 Technology Square, Cambridge, MA 02139, USA; Off No: +1 857-268-7104, Email IDs: [laraphds@gmail.com](mailto:laraphds@gmail.com), LRonsard@mgh.harvard.edu

**Supplemental Figures:**


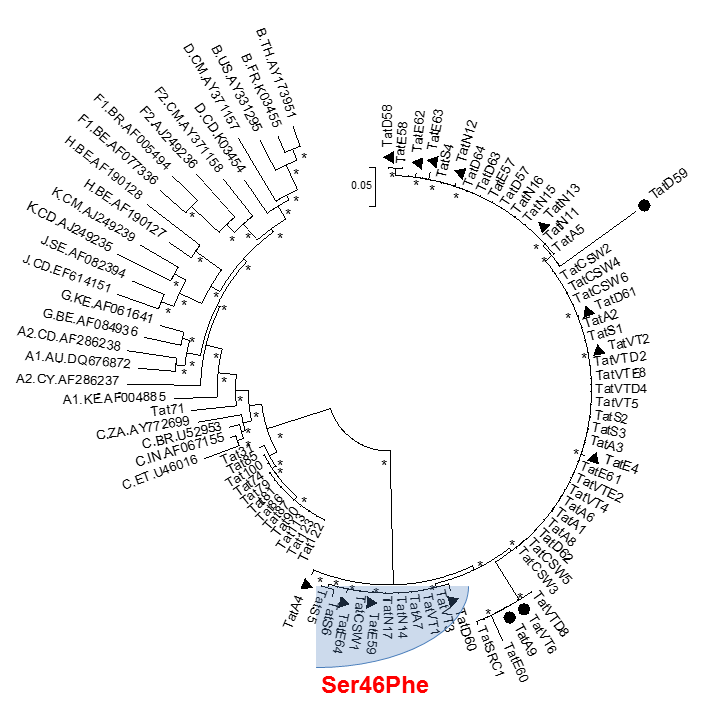


Figure S1. **HIV-1 subtyping of Tat variants.** Phylogenetic tree of Tat variants with M group reference subtypes (A to K including A1, A2, F1, and F2). Each reference sequence was labelled with subtype, followed by the country of isolation and accession number. Filled triangles represent C variants, filled circles represent B/C variants. Mega Version 6 is used for construction of phylogenetic tree with the bootstrap probability (>60%, 1,000 replicates) indicated with an asterisk (*) at the corresponding nodes of the tree and the scale bar represents the selection distance of 0.05 nucleotides per position in the sequence.


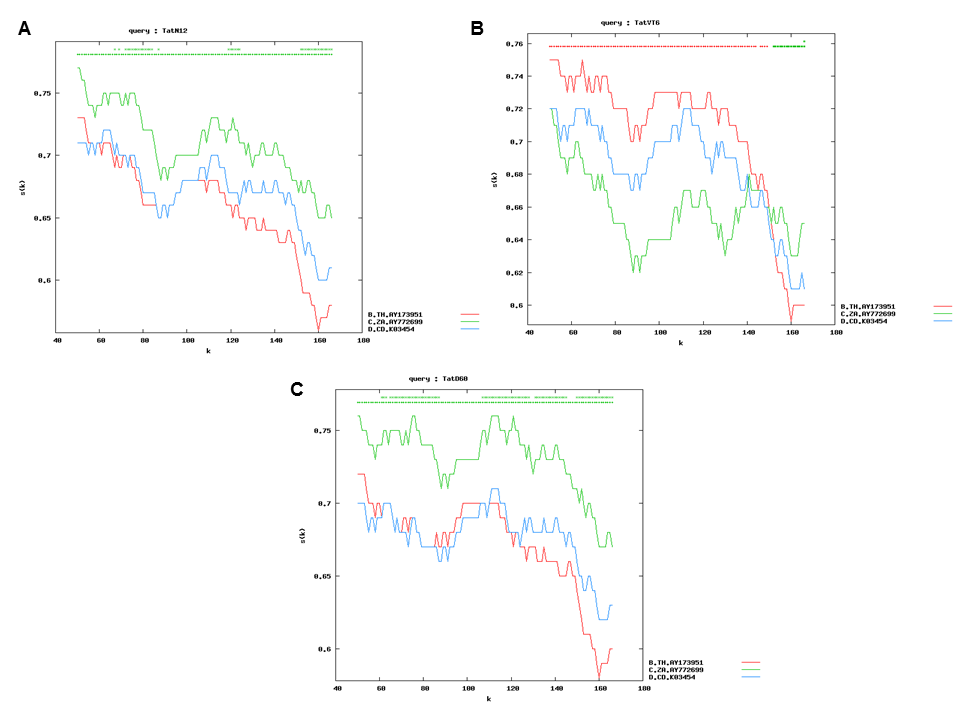


Figure S2. **Recombination events in Tat variants.** A) TatN12 is a representative of Tat C variant. B) TatVT6 is a representative of Tat B/C recombinant. C) TatD60 is a representative of Tat C variant with Ser46Phe mutation. The green line represents subtype C and the red line represents subtype B. RIP (Recombinant Identification Program) is used to identify the recombination events in the Tat variants with a confidence threshold 90% and a window size of 100.


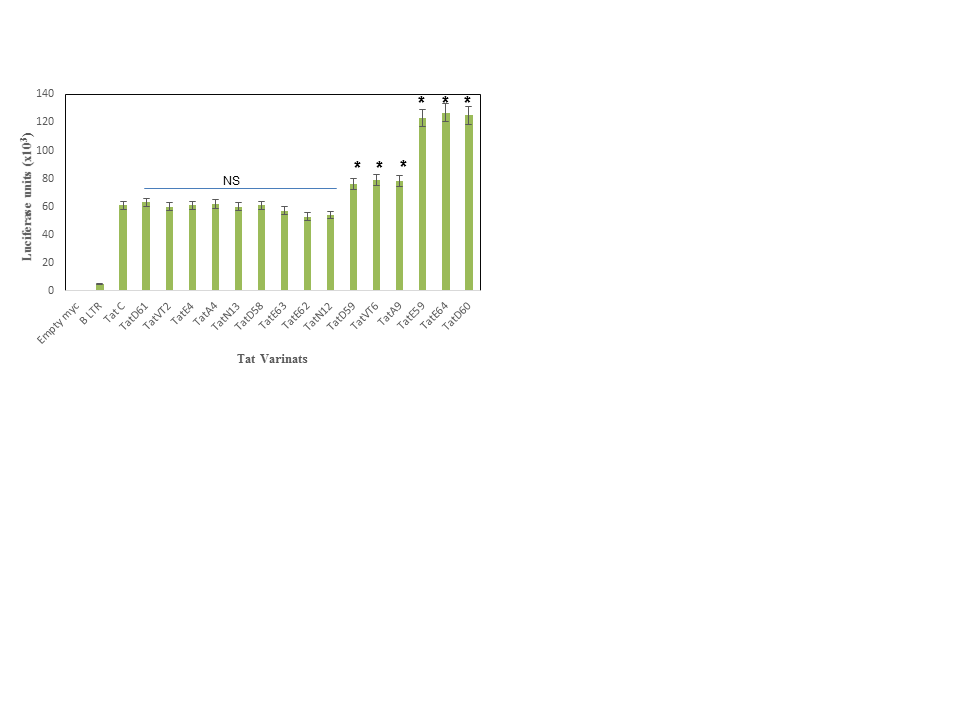


Figure S3. **HIV-1 LTR transactivation by Tat variants**. Tat variants from HIV-1 infected individuals (n=15) were aligned with wild-type Tat C (C.IN.93.93IN905). HEK293 cells were co-transfected with pCMV-myc Tat variants and pGL3-Luc subtype B LTR. After 24 hours of transfection, cells were harvested and lysed and luciferase activity was measured. The relative transactivation was expressed as mean luciferase units. Wild-type TatC was used as reference Tat for comparison. Empty myc vector was used as a control. Subtype B LTR was used as loading control. Luciferase activity of 15 Tat variants and wild-type Tat C normalized to empty pCMV-myc vector.


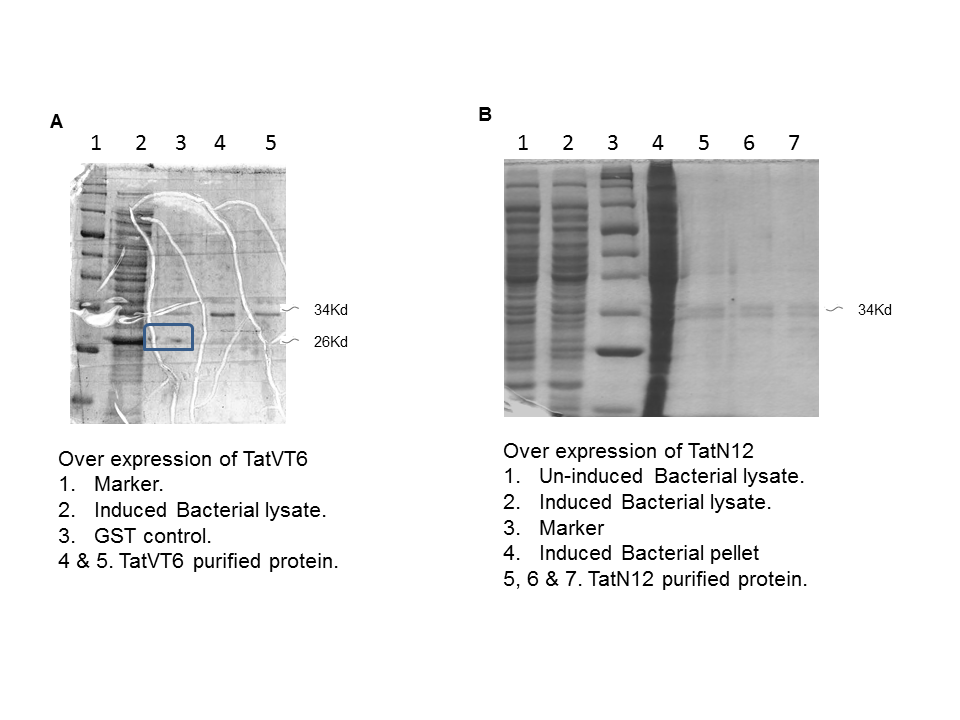


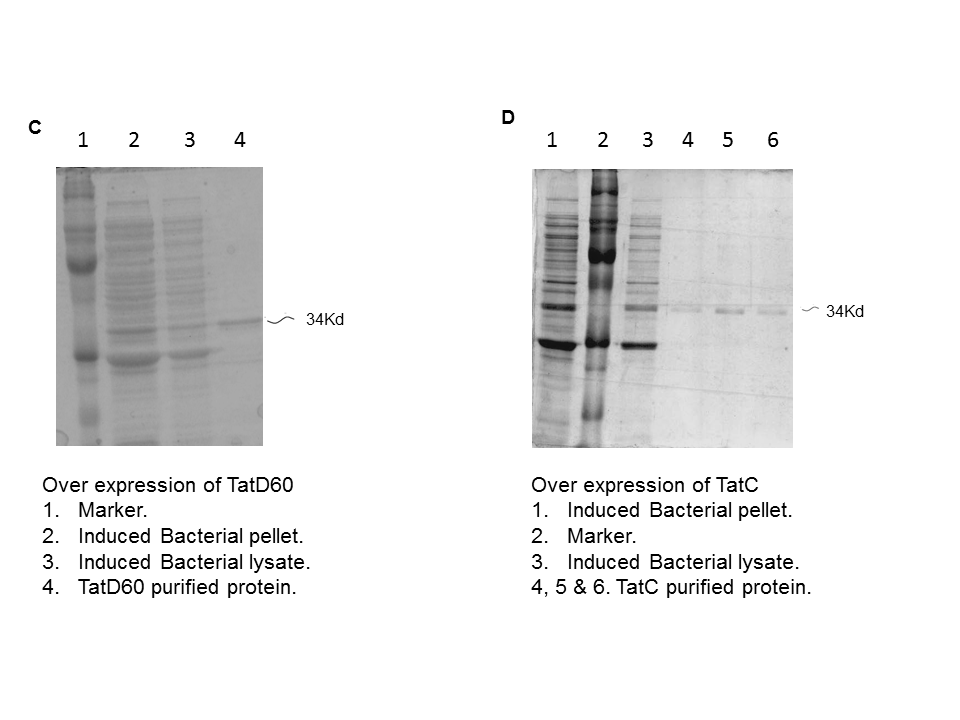


Figure S4. **Over expression of Tat proteins on SDS PAGE followed by Coomassie brilliant blue staining.** A) Over expression of TatVT6. B) Over expression of TatN1. C) Over expression of TatD60. D) Over expression of TatC.

**Supplemental Tables:**

| **Energy components*^a^*** | **TatC-TAR** | | | **TatD60-TAR** | | |
| --- | --- | --- | --- | --- | --- | --- |
|  | **Mean**  **kcal/mol** | **STD** | **SEM** | **Mean**  **kcal/mol** | **STD** | **SEM** |
| ∆E_vdW_ | -52.7207 | 11.13 | 0.1574 | -94.1864 | 7.7128 | 0.1091 |
| ∆E_elect_ | -3219.8769 | 93.87 | 1.3275 | -3122.7773 | 108.1655 | 1.5297 |
| ∆E_GB_ | 3212.9974 | 93.33 | 1.3200 | 3149.0486 | 105.9988 | 1.4990 |
| ∆E_SURF_ | -7.5481 | 1.08 | 0.0153 | -12.5794 | 0.7743 | 0.0110 |
| ∆G_gas_ | -3272.5976 | 96.60 | 1.3662 | -3216.9637 | 110.3264 | 1.5603 |
| ∆G_solv_ | 3205.4493 | 92.80 | 1.3124 | 3136.4692 | 105.9368 | 1.4982 |
| ∆G_bind_ | -67.1484 | 9.6599 | 0.1366 | -80.4945 | 9.2325 | 0.1306 |

**Table S1**. Binding free energies of wild type TatC-TAR and TatD60-TAR complex calculated by Generalized Born (GB) methodology. [*^a^*The binding energy components are calculated from ∆G = ∆G_complex_− ∆G_receptor_− ∆G_ligand_. ∆E_vdW_ - van der Waals energy contribution; ∆E_elect_ – electrostatic energy contribution; ∆E_GB_ = Generalized Born field potential; ∆G_bind_ – free energy of binding; ∆E_SURF_ – non-polar contribution of solvation free energy; ∆G_gas_ =∆E_vdW_ + ∆E_elect_ ; ∆G_solv_ = ∆E_GB_ + ∆E_SURF_; ∆G_bind_ = ∆G_gas_ - ∆G_solv_ ].

| **Residues and positions** | **TatD60** | | **Tat C** | |
| --- | --- | --- | --- | --- |
|  | **Total (kcal/mol)** | **STD** | **Total (kcal/mol)** | **STD** |
| CYS 25 | -0.141 | 7.312 | 0.006 | 8.941 |
| TYR 26 | -0.721 | 6.22 | 0.012 | 6.805 |
| CYS 27 | 0.07 | 7.202 | 0.038 | 10.324 |
| LYS 28 | -1.64 | 18.34 | -0.231 | 31.124 |
| TYR 29 | -1.363 | 7.719 | 0.034 | 8.547 |
| CYS 30 | -0.102 | 6.542 | 0.121 | 8.091 |
| SER 31 | 0.194 | 9.016 | 0.157 | 9.678 |
| TYR 32 | -0.817 | 5.52 | -0.202 | 6.022 |
| HIS 33 | -2.207 | 7.163 | -0.832 | 11.155 |
| CYS 34 | -0.432 | 6.866 | -0.002 | 9.131 |
| LEU 35 | -6.447 | 9.452 | -0.189 | 5.97 |
| VAL 36 | -2.816 | 7.436 | 0.022 | 5.811 |
| CYS 37 | 0.24 | 9.246 | 0.009 | 5.557 |
| PHE 38 | 0.015 | 6.78 | -0.041 | 7.117 |
| GLN 39 | 0.034 | 10.313 | 0.025 | 9.709 |
| THR 40 | 0.036 | 8.72 | 0.091 | 9.069 |
| LYS 41 | -0.123 | 17.023 | -0.12 | 39.084 |
| GLY 42 | 0.018 | 5.765 | 0.026 | 5.146 |
| LEU 43 | -0.025 | 6.549 | -0.004 | 7.116 |
| GLY 44 | -0.087 | 4.289 | -0.129 | 7.253 |
| ILE 45 | -0.119 | 6.108 | -0.313 | 8.294 |
| **PHE/SER 46** | **0.052** | **5.117** | **-4.934** | **8.885** |
| **TYR 47** | **0.024** | **6.066** | **-3.909** | **8.186** |
| **GLY 48** | **0.055** | **5.861** | **-5.291** | **5.452** |
| ARG 49 | -12.07 | 15.687 | -16.524 | 17.477 |
| LYS 50 | -0.136 | 19.655 | -0.581 | 26.573 |
| LYS 51 | -5.266 | 18.739 | -7.876 | 14.652 |
| ARG 52 | -4.296 | 23.996 | -10.619 | 16.843 |
| ARG 53 | -13.688 | 12.174 | -6.192 | 24.679 |
| **GLN 54** | **-8.058** | **9.557** | **0.065** | **7.828** |
| ARG 55 | -0.992 | 27.156 | -0.27 | 20.04 |
| ARG 56 | -0.711 | 18.819 | -0.514 | 19.331 |
| SER 57 | -1.691 | 9.046 | -0.052 | 10.242 |
| ALA 58 | -1.704 | 6.009 | -1.211 | 4.866 |
| PRO 59 | 0.177 | 5.576 | 0.153 | 5.425 |
| GLN 60 | -0.969 | 7.626 | 0.016 | 7.593 |
| SER 61 | 0.077 | 6.631 | 0.03 | 8.069 |
| SER 62 | 0.117 | 9.306 | 0.011 | 5.746 |
| GLU 63 | 0.51 | 24.818 | 0.395 | 23.479 |
| ASP 64 | 0.688 | 42.806 | 0.317 | 29.573 |
| HIS 65 | -0.163 | 8.69 | -0.048 | 7.857 |
| GLN 66 | -0.23 | 10.789 | 0.084 | 9.822 |
| **ASN 67** | **-8.708** | **6.229** | **0.181** | **7.948** |
| LEU 68 | -2.46 | 6.421 | -0.964 | 7.726 |
| ILE 69 | -0.599 | 8.765 | -0.633 | 7.124 |
| SER 70 | 0 | 9.924 | 0.296 | 11.066 |
| LYS 71 | 1.557 | 34.382 | -0.792 | 42.649 |

**Table S2.** Residue-wise binding energy contribution for TatC-TAR and TatD60-TAR interactions.

| **Sample** | **Age**  **(year)** | **Sex** | **Mode of**  **transmission** | **Positive since detection** | **Viral load after 6 months of treatment [copies/ ml]** | **Initial status of patient** | **Initial CD4**  **count** | **CD4**  **count after 6 months of treatment** | **Predicted Subtype** | **Group based on this study** |
| --- | --- | --- | --- | --- | --- | --- | --- | --- | --- | --- |
| TatD61 | 30 | F | Heterosexual | 2008 | 64 | ART +ve | 376 | 443 | C | 1 |
| TatVT2 | 4 | M | Vertical | 2007 | <50 | ART +ve | 727 | 856 | C | 1 |
| TatE4 | 6 | M | Vertical | 2008 | 72 | ART +ve | 972 | 984 | C | 1 |
| TatA4 | 30 | M | Heterosexual | 2004 | <50 | ART +ve | 351 | 489 | C | 1 |
| TatN13 | 39 | F | Heterosexual | 2010 | <50 | ART +ve | 104 | 234 | C | 1 |
| TatD58 | 28 | F | Heterosexual | 2008 | <50 | ART +ve | 257 | 245 | C | 1 |
| TatE63 | 6 | M | Vertical | 2008 | <50 | ART +ve | 652 | 789 | C | 1 |
| TatE62 | 7 | M | Vertical | 2008 | <50 | ART +ve | 572 | 624 | C | 1 |
| **TatN12** | **27** | **F** | **Heterosexual** | **2010** | **<50** | **ART +ve** | **226** | **350** | **C** | **1** |
| TatD59 | 31 | F | Heterosexual | 2008 | 135 | ART +ve | 344 | 324 | B/C | 2 |
| **TatVT6** | **6** | **M** | **Vertical** | **2006** | **182** | **ART +ve** | **1048** | **976** | **B/C** | **2** |
| TatA9 | 35 | M | Heterosexual | 2006 | 157 | ART -ve | 447 | 434 | B/C | 2 |
| TatE59 | 7 | F | Vertical | 2008 | 171 | ART +ve | 687 | 521 | C | 3 |
| TatE64 | 10 | M | Vertical | 2008 | 278 | ART +ve | 458 | 342 | C | 3 |
| **TatD60** | **33** | **F** | **Heterosexual** | **2008** | **346** | **ART +ve** | **336** | **214** | **C** | **3** |
| **Note:**  ART +ve = Anti-Retroviral Therapy positive  ART -ve = Anti-Retroviral Therapy negative  F=Female  M=Male | | | | | | | | | | |

**Table S3.** Clinical data of Tat variants from HIV-1 infected patients from North India.
